# A comparative study for the inactivation of multidrug resistance bacteria using dielectric barrier discharge and nano-second pulsed plasma

**DOI:** 10.1038/srep13849

**Published:** 2015-09-09

**Authors:** Ji Hoon Park, Naresh Kumar, Dae Hoon Park, Maksudbek Yusupov, Erik C. Neyts, Christof C. W. Verlackt, Annemie Bogaerts, Min Ho Kang, Han Sup Uhm, Eun Ha Choi, Pankaj Attri

**Affiliations:** 1 Plasma Bioscience Research Center/Department of Electrical and Biological Physics, Kwangwoon University, Seoul, Korea 139-701; 2 Research Group PLASMANT, Department of Chemistry, University of Antwerp, Universiteitsplein 1, 2610 Antwerp, Belgium

## Abstract

Bacteria can be inactivated through various physical and chemical means, and these have always been the focus of extensive research. To further improve the methodology for these ends, two types of plasma systems were investigated: nano-second pulsed plasma (NPP) as liquid discharge plasma and an Argon gas-feeding dielectric barrier discharge (Ar-DBD) as a form of surface plasma. To understand the sterilizing action of these two different plasma sources, we performed experiments with *Staphylococcus aureus* (*S. aureus*) bacteria (wild type) and multidrug resistant bacteria (Penicillum-resistant, Methicillin-resistant and Gentamicin-resistant). We observed that both plasma sources can inactivate both the wild type and multidrug-resistant bacteria to a good extent. Moreover, we observed a change in the surface morphology, gene expression and β-lactamase activity. Furthermore, we used X-ray photoelectron spectroscopy to investigate the variation in functional groups (C-H/C-C, C-OH and C=O) of the peptidoglycan (PG) resulting from exposure to plasma species. To obtain atomic scale insight in the plasma-cell interactions and support our experimental observations, we have performed molecular dynamics simulations to study the effects of plasma species, such as OH, H_2_O_2_, O, O_3_, as well as O_2_ and H_2_O, on the dissociation/formation of above mentioned functional groups in PG.

In the early 1970s, physicians believed that all bacterial infections were treatable, but their expectations were shattered when pathogens developed resistance to multiple antibiotics, such as *Pseudomonas aeruginosa, Mycobacterium tuberculosis, Staphylococcus aureus and Streptococcus pneumoniae*^1^,^2^,^3^,^4^. As a result, there was an increased interest in studying the molecular mechanisms to understand the antimicrobial resistance of such bacteria in order to identify novel drug targets. However, effective chemotherapeutic agents have not yet been found. Among all multidrug resistant bacteria, *Staphylococcus aureus* (*S. aureus*) is perhaps of greatest concern because it has the capacity to cause disease through a diverse array of life-threatening infections^1^,^5^. In the early 1940s, penicillin was introduced to treat patients affected by *S. aureus*. Nevertheless, in 1942, hospitals had encountered penicillin-resistant staphylococci, and up to the late 1960s, more than 80% of all hospitals had isolated staphylococcal strains resistant to penicillin^1^,^6^. In 1961, methicillin was introduced as a drug against *S. aureus*. However, some infections were soon found to be a result of *methicillin-resistant S. aureus*^1^,^7^. Moreover, *gentamicin-resistant S. aureus* has also been clinically isolated, and its mechanism has been observed to be different from that of penicillin and methicillin^8^. Since chemical drugs do not provide a proper solution to inactivate *S. aureus*, there is an essential need to implement treatment other than antibiotics.

Atmospheric pressure non-thermal plasma (APP) provides a means to efficiently and effectively control multidrug resistant microorganisms^9^,^10^. Depending on the sources and discharge conditions, APP is an efficient source of a combination of electronically excited atoms, charged particles (electrons, ions), ozone (O_3_), UV photons and radicals^9^,^10^,^11^,^12^,^13^,^14^,^15^,^16^,^17^,^18^,^19^. APP has shown great promise for use to sterilize medical products and packaging materials since it effectively inactivates bacteria and bacterial spores, besides of material modification^9^,^10^,^11^. Many studies have investigated plasma-based sterilization of immobilized microorganisms on a material surface under dry condition^20^,^21^,^22^. Although reactive species and free radicals in the gas phase directly damage microorganisms^20^,^21^,^22^, sterilization under wet conditions is important for a variety of practical applications.

When bacteria are submerged in a liquid, neither electrons nor ions can interact directly with the surface of the bacteria since these electrons or ions are strongly absorbed by the liquid when passing through the gas–liquid interface^23^. A liquid environment provides an additional obstacle for plasma to have a direct interaction with microorganisms, and in addition, a wet environment provides good living and growth conditions for microorganisms. Consequently, plasma-induced UV irradiation and reactive species (RS), including reactive oxygen species (ROS) and nitrogen species (RNS), penetrate the liquid and interact with the microorganism cells. The RS created by plasma influence the cell signaling pathways of many biological systems (from bacteria to mammalian cells)^24^. However, our understanding of the interaction between plasma and bacteria and their action mechanism is still limited.

Hence, we investigate two types of APP systems in this work: one is nano-second pulsed plasma (NPP) as liquid discharge plasma and the other is an Argon gas feeding dielectric barrier discharge (Ar-DBD) as surface plasma. We use *S. aureus* bacteria (wild type) and multidrug-resistant bacteria (*Penicillum-resistant S. aureus* (PRSA), *Methicillin-resistant S. aureus* (MRSA) and *Gentamicin-resistant S. aureus* (GRSA)) to understand the plasma activity. Moreover, we have studied the RS both inside the bacteria and in saline solution. A scanning electron microscope (SEM) was used to study the changes in the surface morphology of the bacteria. In addition, we studied the action of the plasma on the gene expression and β-lactamase activity. Furthermore, we used X-ray photoelectron spectroscopy (XPS) to investigate the variation in the functional groups (C-H/C-C, C-OH and C=O) of peptidoglycan (PG) (outer part of cell membrane). Moreover, we studied the interaction of the reactive (i.e., O, OH, O_3_ and H_2_O_2_) as well as non-reactive (i.e., O_2_, H_2_O) plasma species with PG of *S. aureus*, using reactive molecular dynamics (MD) simulations.

## Results

Many kinds of NPP and DBD plasma sources have been reported in the literature to inactivate different types of bacteria^9^,^10^,^11^,^20^,^21^,^22^,^23^. However, the action that these plasma sources have on drug resistance bacteria, and its underlying mechanism, is still not fully understood. For this study, we have used an NPP to its 4^th^ discharge because we observed in our earlier work that the flux of RS created by the NPP increases as the number of discharges increases^13^. For the Ar-DBD, we studied the RS at different time intervals and then optimized the time of the treatment to 5 min (data not shown).

### Analysis of the change in the physical parameters, production of the RS and shock waves generation during plasma exposure

**Figure 1:**
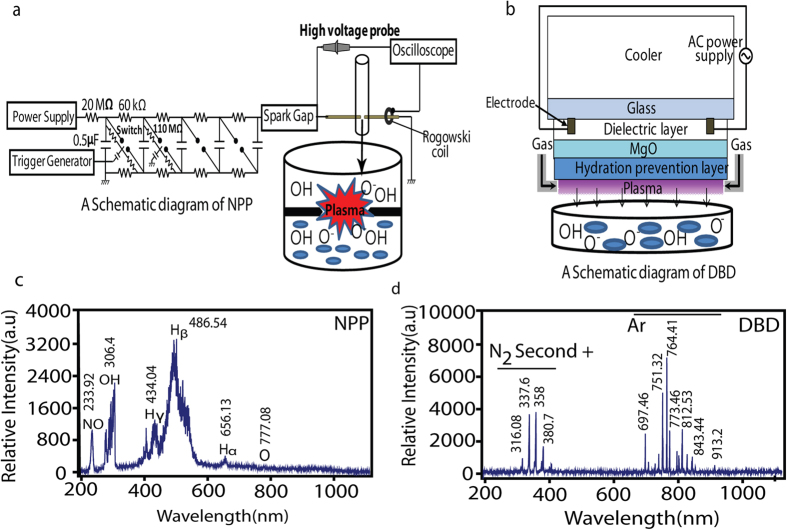
(**a**) Schematic diagram of NPP, (**b**) Schematic diagram of Ar-DBD, (**c**) Measurement of the optical emission spectra of NPP and (**d**) Measurement of the optical emission spectra of Ar-DBD.

Figure 1a,b represents the schematic diagram of the NPP and Ar-DBD plasma, respectively. The breakdown voltage and breakdown current for the NPP is 6 kV and 0.7 kA, respectively, with the energy of 0.06 J/discharge. Whereas, for Ar-DBD the V_rms_ is 0.6 kV and I_rms_ is 14 mA with energy of 1.38 J/s. Waveform of the NPP and Ar-DBD are shown in Figure S1a,b. Figure 1a, shows that the NPP plasma creates the RS inside the saline. On the other hand, the Ar-DBD generates surface plasma, and then, the RS created on the saline surface or on the plasma surface due to gas or atmospheric conditions penetrate into the saline, as seen in Fig. 1b. We investigated the OES spectra to further understand the RS created by the plasma (Fig. 1c,d). We detected an emission spectrum between 200 and 1100 nm from the NPP in distilled water. An atomic oxygen excitation line is clearly observed at 777.08 nm due to the 3p^5^P → 3s^5^S transition, as well as H_α_ line ~656 nm, H_β_ line ~486 nm, H_γ_ line ~434 nm and OH line ~309 nm resulting from A^2^Σ^+^ → X^2^Π (Fig. 1c). In the case of the Ar-DBD, we observed weak lines belonging to the molecular NO β, γ system between 200 and 250 nm. The most intensive emission lines in the Ar-DBD are the second positive system N_2_ emission bands (300–440 nm, C^3^Π_u_ → B^3^Π_g_) and the argon (Ar I) emission (4p → 4s) lines in the spectral region from 690 to 860 nm (Fig. 1d). Furthermore, we measured the temperature and pH of the saline solution after treatment. We observed that the temperature of solution increases for both plasma devices after treatment at up to ~28 °C, and for NPP, it is ~21 °C, which is much below the critical temperature for biological organisms (Figure S1c). While the pH substantially decreases for the Ar-DBD after treatment for 5 min to ~4.2, it remains at ~5.3 for NPP (Figure S1d). This indicates that the change in temperature and pH is greater for the Ar-DBD treatment as compared to the NPP treatment.

**Figure 2:**
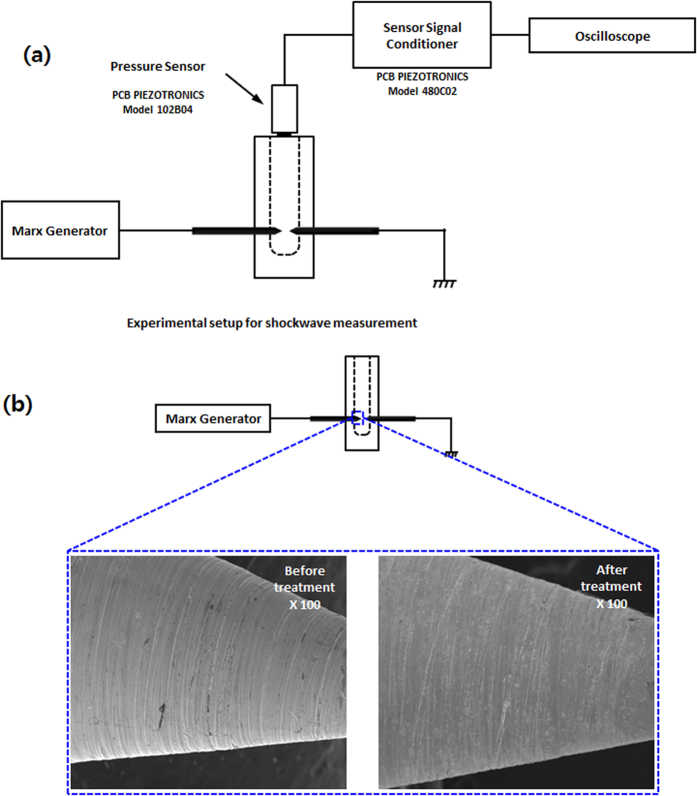
(**a**) Schematic diagram for shockwaves analysis in NPP and (**b**) SEM images of electrode before and after discharge.

In addition, we performed a chemical analysis to test the radicals^15^,^19^ produced inside the saline solution during the NPP and Ar-DBD exposure, as shown in Figure S2. We observed a higher amount of OH^•^ for DBD after 5 min than seen after the 4^th^ NPP discharge, as shown in Figure S2. While in the presence of the Trolox, we still observed a higher presence of OH^•^ for the DBD than for the NPP. Similar results were observed for the H_2_O_2_ and NO radicals where DBD has more production than NPP. If we compare Figures S2a, c we observe that in the presence of Trolox and cPITO, the amount of H_2_O_2_ and NO deceases significantly, respectively. In the NPP shock wave is another factor that provides additional action during the treatment as compared with Ar-DBD. Therefore, we have checked the shock wave by analyzing the rising time of piezoelectric pressure gauge for measuring pressure as per standard protocol provided by manufacturing company, the setup is shown in Fig. 2a. We observed the shock waves of 6.4 atm (4912 torr) at 7 cm above the electrode in 1.1 ms propagation time, using piezoelectric pressure gauge as shown in Figure S3. If we placed the senor more near the electrode than we can’t observe the proper signal due to interference of current and voltage peaks (data not shown). Latter, we checked the SEM images of electrode before and after discharges, as shown in Fig. 2b. This shows that there is no degradation of electrode during the treatment.

### Inactivation and the changes in morphology of multidrug resistant *S. aureus* after treatment

**Figure 3:**
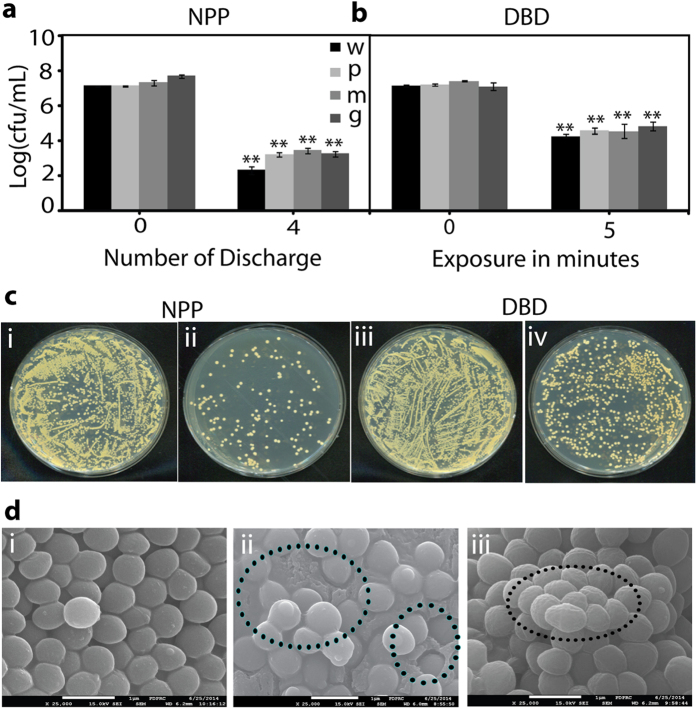
(**a**) Inactivation of wild type and multidrug resistance bacterial strains of *S. aureus* by NPP, (**b**) Inactivation of wild type and multidrug resistance bacterial strains of *S. aureus* by Ar-DBD, (**c**) Colony of *S. aureus* (wild type) after NPP and Ar-DBD treatment and (**d**) SEM image of the *S. aureus* (wild type) (i) after NPP (ii) and Ar-DBD treatment (iii). All values are expressed as ± SD in triplicates. Students’t-test was performed to control (* denotes P < 0.05 and ** denotes P < 0.01).

 The results for inoculated colonies of *S. aureus* (wild type) and multidrug resistant bacteria are shown in Figure 3a,b. We have taken 10^7^ CFU/ml (i.e., colony forming unit per ml) for all strains, and after the 4^th^ discharge of NPP, 10^2^ CFU/ml remained for wild-type *S. aureus*, and 10^3^ CFU/ml survived for multidrug resistance bacteria (PRSA, MRSA and GRSA). This means that after the NPP plasma discharge, log 4–5 bacteria were inactivated (Fig. 3a) while only log 3 bacteria were inactivated for Ar-DBD plasma, which indicates that 10^4^ CFU/ml bacteria survived (Fig. 3b). Figure 3c shows a colony of *S. aureus* (wild type) on an agar plate following NPP and Ar-DBD treatment. We observed a higher reduction in bacterial colonies for NPP plasma treatment than for Ar-DBD treatment.

SEM images of *S. aureus* were taken before and after NPP and DBD treatment (Fig. 3d). After the NPP treatment, the bacteria endured a transition from initially smooth surfaces to crushed and ruptured surfaces. After treatment with Ar-DBD, the bacterial surfaces become more wrinkled, which was not observed for the control samples (where Ar gas was used to treat bacteria in saline without plasma). The change in the morphology of the cell wall of the bacteria was considered to be unfavorable for survival. SEM images of the multidrug resistance bacteria are shown in Figure S4.

### Change in intracellular ROS, gene expression and β-lactamase activity

**Figure 4:**
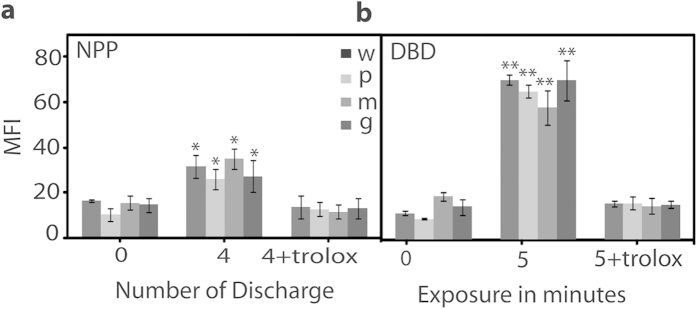
Intracellular ROS estimation after NPP and Ar-DBD treatment and in the presence of ROS scavenger trolox. All values are expressed as (MFI) and ±SD in triplicates. Students’t-test was performed to control (* denotes P < 0.05 and ** denotes P < 0.01).

ROS play a very important role in the bactericidal activity. Due to the increase in extracellular ROS or stress, the intracellular ROS also increased. Hence, we interpreted enhanced intracellular ROS in case of the NPP treatment relative to the DBD treatment as observed in Figures 4a,b. The intracellular ROS for Ar-DBD increased 3 times relative to the control sample. We can predict that extracellular ROS or RNS radicals, which have a short life time or long life time, play a crucial role in increasing the intracellular ROS of the multidrug resistant and wild type *S. aureus* bacteria due to stress. However, we observed less intracellular stress in the presence of Trolox, as shown in Fig. 4.

β-lactam antibiotics are penicillin-binding proteins (PBPs), and these enzymes are responsible for cross-linking the PG in the bacterial cell wall^25^. They might further target RS generated by the NPP and DBD plasma. The action of the β-lactam antibiotics can be resisted by *Staphylococci*, due to the presence of the mecA gene, which encodes a PBP, PBP2a, which has a reduced affinity for β-lactams^26^,^27^. The methicillin-resistant strains of other staphylococcal species contain gene mecA^27^,^28^,^29^. Moreover, two regulatory genes, mecI and mecR1 are located upstream of mecA and control the expression of mecA. These regulatory genes encode a mecA repressor protein and a signal transducer protein, respectively^30^,^31^. mecRI is activated upon contact with β-lactam, which is equivalent to an inducing factor with MRSA, and its signal binds to the promoter region of mecA and transduces to mecI, which suppresses transcription, and thus the suppression is cleared. Since the femA gene is involved in the components of the cell wall of *S. aureus*, it therefore mediates the effect on drug sensitivity and is involved in methicillin resistance^27^,^28^,^29^,^32^.

**Figure 5:**
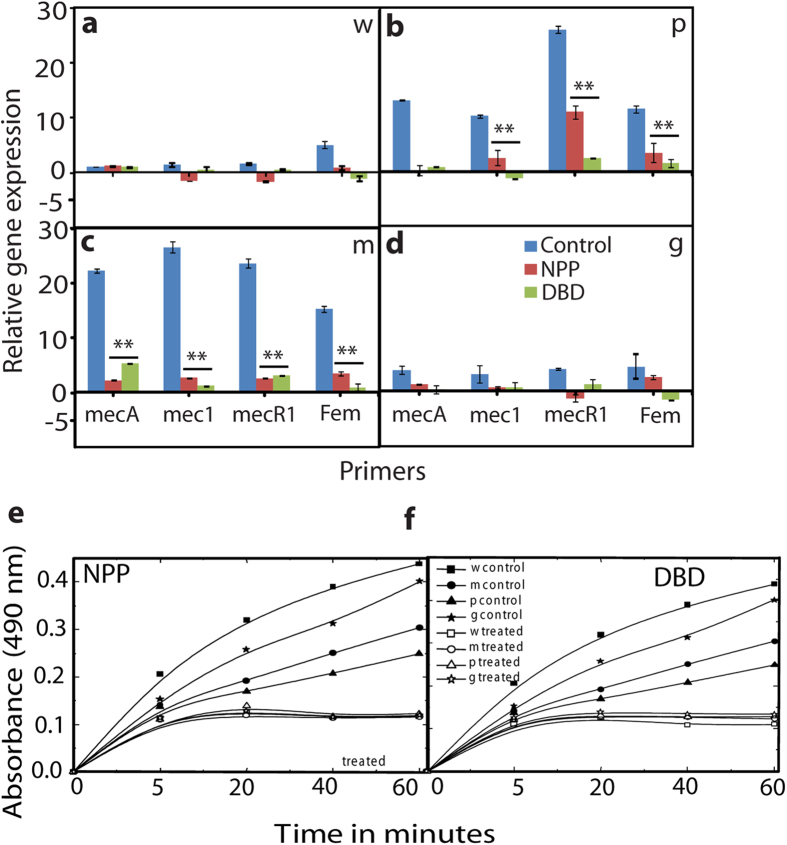
Gene expression analyses after NPP and Ar-DBD treatment (a) Wild type *S. aureus*, (b) PRSA, (c) MRSA, (d) GRSA, (e) β-lactamase activity of NPP and (f) β-lactamase activity of Ar- DBD. All values are expressed as (MFI) and ± SD in triplicates. Students’t-test was performed to control (* denotes P < 0.05 and ** denotes P < 0.01).

A quantitative real time polymerase chain reaction (Q-PCR) analysis of the level of mecA, mecI, mecRI and femA gene in the MRSA as well as wild strains was performed after exposure to NPP and DBD plasma, as shown in Fig. 5a–d. Fig. 5a–d, depicts the inhibition for resistant expressions of mecA, mecI, mecRI and femA gene in comparison with the untreated sample. In addition, the NPP and DBD exposure inhibited the mecRI induced by mecR. A similar phenomenon was exhibited by femA, which is known to be related to the composition of the cell wall. Although untreated wild type strain genes such as mecA, mecI, mecRI are not prompt. Thus, our Q-PCR analysis results indicate that RS generated by the NPP and DBD can inhibit the gene expression related to bacterial resistance. To further confirm this result, we studied the β-lactamase activity in wild and in multidrug resistant bacteria. We observed that as the time increases, the β-lactamase activity also increases from 5 to 60 min for *S. aureus* (wild type) and multidrug resistant bacteria (PRSA, MRSA and GRSA), as shown in Fig. 5e,f. The activity is highest for the wild type, followed by that for the MRSA, PRSA and GRSA without plasma treatment. After plasma treatment with both NPP and Ar-DBD plasma, the activity was reduced significantly as a function of time. After 5 min of treatment, β-lactamase activity was slightly prominent in case of all bacteria, but after 20 min, the activity decreased more and then became constant. However, the decreasing trend is the same for both plasma treatments with a slight variation.

### Analysis of the plasma species generated by the NPP and Ar-DBD on the bacterial cell wall using XPS

We conducted XPS measurements to study the influence of the NPP and Ar-DBD plasma treatment on the bacterial PG, which is the outer part of the cell wall, as a system of plasma–bacteria interactions for wild-type and multidrug resistant bacteria. The results from the XPS measurements are summarized in Figures S5–S12. In this study, we investigated the changes in carbon (C 1s) and oxygen (O 1s) after the plasma treatment. All XPS peaks were examined according to those reported in prior studies^13^,^14^,^33^,^34^.

The C 1s and O 1s core level spectrum of the *S. aureus* (wild type) and multidrug resistant bacteria (PRSA, MRSA and GRSA) are shown in Figures S5-S12. The binding energies were corrected by taking the C 1s core level at 284.6 eV for all samples^13^,^14^,^35^. The carbon 1s XPS spectra show signals at 284.6, 286.02, 287.06 and 289.04 eV, respectively, which correspond to the C–C/C-H, C–O and C=O functional groups of bacteria in wild type and in drug resistant bacteria. On the other hand, the O 1s spectrum of the bacteria (wild type and multidrug resistant) revealed peaks at 531.0 and 532.1 eV, corresponding to the C=O and C-OH groups, respectively, although we did not observe any additional peaks for C 1s and O 1s after NPP treatment on wild type, PRSA, MRSA and GRSA strains. Similar results could be observed after treating all bacteria with Ar-DBD, and the composition percentage of carbon and oxygen changed significantly after treatment, as shown in Table 1. Before treatment the C content is 44.59% and that of O is 39.97%. However, after treatment with NPP, the O content is 52.51%, and after treatment with DBD, the O content is 45.48%. Similar results could be observed for the multidrug resistant bacteria, where the percentage of C decreased and the percentage of O increased after treatment relative to without treatment.

### Study of ROS action on peptidoglycan (PG) using MD simulations

**Figure 6:**
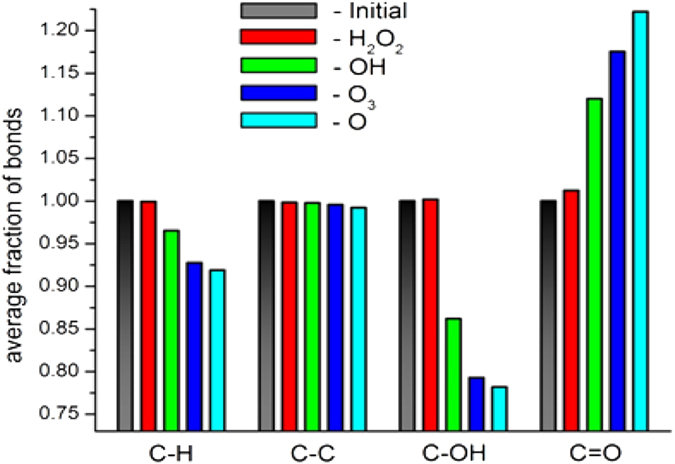
Average fraction of important bonds of the PG after impact of various plasma species. Note that the values are calculated/averaged from 50 independent simulations for each incident species.

To better understand this action, we performed reactive MD simulations, which allow to investigate processes on the atomic level. We investigated the action of the plasma species on PG of *S. aureus* (including wild type, PRSA, MRSA and GRSA). PG acts as a model system for the interaction between plasma species and bacteria, as reported earlier^36^,^37^. The reactive MD simulations are carried out using the ReaxFF glycine-force field^36^,^37^,^38^. A detailed description of our reactive MD simulations used in this work is given in our previous studies^36^,^37^. Our simulation results show that the ROS, i.e., O, OH, O_3_ and H_2_O_2_, are found to break bonds that are structurally important in the murein (or PG), which results in structural damage of the bacterial cell wall. In contrast, no influence was observed for the non-RS, such as O_2_ and H_2_O. Figure 6 shows the average fractions for the C-H, C-C, C-OH and C=O bonds after the impact of O, OH, O_3_ and H_2_O_2_. An overall decrease is observed in the number of C-H, C-C and C-OH bonds after exposure to the plasma species while the number of C=O bonds increases. For the above-examined species, the O radical was found to be more effective for bond breaking and formation than the other plasma species. These results support our XPS results in that the %O content increased due to plasma treatment.

## Discussion

Plasma medicine and sterilization are two main biomedical applications of atmospheric pressure non-thermal plasma. In the present work, we have discussed the efficiency of the plasma (NPP and Ar-DBD) on the multidrug resistance bacterial inactivation and the possible mechanism thereof.

### OES and Q-PCR measurement analysis

OES measurement shows that many radicals are generated in gas phase for DBD and in liquid state for NPP. Many of these species have short life time and can’t detect in solution, but these radical species have strong influence on bacteria inactivation. Hence using the OES we can predict the role of short lived radicals generated during plasma exposure on bacterial inactivation. In Fig. 3, inoculated colonies of *S. aureus* (wild type and multidrug resistant) bacteria were killed by NPP and Ar-DBD. To further understand this mechanism, we studied the change in the intracellular ROS after plasma treatment. We observed that intracellular ROS concentrations doubled after the treatment with NPP and exceeded more than three times its initial value after the treatment with DBD, as a result of the stress created by the extracellular ROS.

Penicillin binding protein (PBP2A) containing MRSA which has low affinity to β-lactam antibiotics due to the presence of the mecA gene, which is regulated by mecI and mecR1^29^,^30^,^31^. Although mecR1 and mecI were identified as the regulatory element for the production of PBP2A^27^. In this study RS generated by the NPP and Ar-DBD were shown to be effective against MRSA strains which carried both the β-lactamase as well as antibiotic resistance mecA gene. On the other hand, PBPs related genes in GRSA are not highly prompted as compared with MRSA and PRSA. This indicates that diversity of antibiotic resistance caused by different regulatory gene besides of mecA.Therefore Q-PCR study of related PBPs such as mec, mecI, mecR1 and femA have inhibitory effects after exposure with NPP and Ar-DBD. Hence, through NPP and Ar-DBD we can inactivate the penicillin binding proteins and regulatory factors that might be affect the peptidoglycan (PG).

### β-lactamase activity and SEM analysis

These results provide evidence that the ROS/RNS play a significant role in bacterial inactivation using NPP and DBD. The bacterial inactivation is greater for NPP than for DBD even though there are more the ROS/RNS for the DBD. If we compare the β-lactamase activity, we can observe that both NPP and DBD resulted in a decrease in activity, which may be due to the inactivation of the β-lactamase enzyme as well as its related gene. These results are consistent with our previous research^19^,^39^,^40^, as well as with results reported by other groups, in the sense that plasma can modify the enzyme structure and reduce the activity^41^,^42^. Moreover, the SEM images show that the NPP treatment crushed many bacterial spores due to shock waves while only the DBD treatment changed the morphology. This shows that NPP, excluding RS and other factors, also plays a major role in producing shock waves to inactivate more bacteria than with DBD.

### XPS and MD simulation results analysis

Furthermore, we used XPS to study the NPP and DBD action on the PG of the bacteria. The XPS data clearly show that the %C content decreased and the %O content increased. This shows that plasma has an oxidative effect. The %C and %O content did not change much for the multidrug resistance bacteria relative to the wild type after plasma treatment, which might be due to a modification that occurs in the PG of resistant bacteria. However, we can conclude that the plasma species created by both plasma sources can oxidize, modify, or damage the bacterial cell wall, which is the main cause for bacterial inactivation. These results are also supported by the fact that the plasma species can damage the cell wall of the bacteria^43^. Furthermore, we performed MD simulation of the PG, as reported earlier^36^,^37^, to verify the action of the ROS (O, OH, O_3_ and H_2_O_2_) on the average fraction of important bonds of the PG (C-H, C-C, C-OH and C=O bonds) to support the result of the XPS data which revealed that %O content increases. The results of the MD simulation indicate that RS (O, OH and O_3_) have a major role in damaging the PG.

### pH role in bacterial inactivation

In our experiments the changes in pH, RNS and ROS are the main parameters responsible for bacterial inactivation of the Ar-DBD. We observed that the pH in saline solution became ~4.2 after 5 min of DBD treatment and ~5.3 after 4^th^ discharge of NPP treatment. Previous studies have focused on the effects of the acidic pH on the antimicrobial activity of plasma^44^. Therefore, the acidification action is tested during our experiment by incubating bacteria (wild type and multidrug resistant) for 2 h in acidic saline, pH controlled using HNO_3_ (Figure S13). We observed no significant antimicrobial effects for both bacteria lines in an acidic environment, which is also supported by previous research^45^. In Fig. 1, we observed NO radicals in solution that can result in the formation of HNO_2_ and HNO_3_, as reported earlier^44^,^45^,^46^,^47^. Hence, the addition of the HNO_3_ and the acidic pH (pH 3 and 4) could not inactivate the multidrug resistant bacteria. These results are also supported by earlier research^45^,^46^,^48^ which observed that additional HNO_3_ and an acidic environment cannot inactivate bacteria. However, the oxidation efficiency of oxidants was also reported to increase in an acidic solution^45^. The Lukes group^43^ reported that nitrogen-based species in Ar-plasma were hypothetically caused by the diffusion of NO to the carrying Ar gas phase, and the presence of NO in solution was also responsible for the inactivation of the bacteria, which had been previously demonstrated^49^. As discussed in our earlier work^19^, that reaction of the NO and O_2_^•−^ can produce a toxic and powerful oxidant, ONOO^−^. In an acidic solution, ONOO^−^ is protonated to form ONOOH, which is a very strong oxidant for biomolecules. P. Lukes *et al*.^50^ recently reported that the formation of ONOOH plays an important role in the antibacterial activity.

### Influence of ozone on bacterial inactivation

Pavlovich *et al*.^51^ suggested that O_3_ plays a major role in bacterial inactivation relative to H_2_O_2_ or acidified nitrite. Thanomsub *et al*.^52^ suggested that 20 mg/h of ozone results in no significant inactivation of *S. aureus* for 5 min for 10^6^ (CFU/ml) whereas for a lower bacterial concentration, the loss is significant. On the other hand, work recently published by Lunov *et al*.^9^ stated that a concentration of 10^6^ (CFU/ml) of *S. aureus* can be inactivated in 60s in a manner similar to air plasma and ozone (400 mg/h) up to 99.99%. Therefore, these results support the idea that an increase in the ozone concentration can increase the inactivation of bacteria, and hence we used the 10^6^ (CFU/ml) concentration of *S. aureus* (wild type and multidrug resistance) in ozone (600 mg/h) in two ways: (1) to treat above the liquid like DBD plasma at a 10 mm distance and (2) to treat inside the liquid (Figure S14). We observed that for 30s, 99% of the bacteria were inactivated for the second type, and 80% of the bacteria were inactivated for the first type of ozone treatment. Our MD simulations also shows that O_3_ has a strong action to decrease the C-H, C-C and C-OH bonds. It was reported earlier that ozone is more soluble and stable in acidic pH^46^. However, our Ar-DBD system can only generate 70 ppm of O_3_ for 5 min in the DBD treatment, as shown in Figure S15. Hence, O_3_ does not have a major contribution in our plasma sources to inactivate bacteria.

### Combined action of H_2_O_2_ and pH on bacterial inactivation

Furthermore, we checked the H_2_O_2_ activity by adding 50 μM of H_2_O_2_ at a pH = 4 (adjusted by HNO_3_), and we observed no significant inactivation of bacteria (Figure S16). Therefore, other radicals relatively contribute more to the total plasma action than H_2_O_2_ and pH. Hence, for the Ar-DBD treatment, the OH, O_2_^•−^, O, NO, ONOO^−^ and ONOOH play an important role in the inactivation of both wild and multidrug resistant *S. aureus* bacteria, as shown in Equations (1–25)^46^,^50^.

### Possible chemical reactions based on the radical species observed in OES measurements and in solution

The small amount of O_3_ is generated in our system and was rapidly decomposed by the 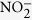, as suggested in an earlier report^46^,^50^.


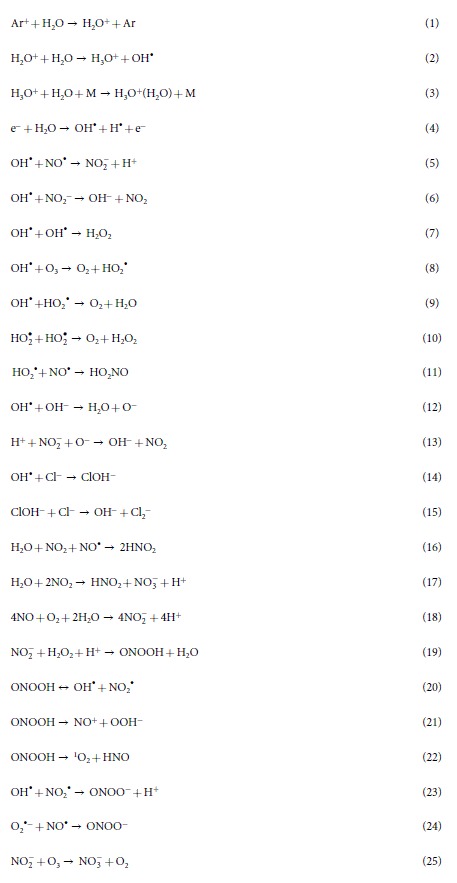


HO_2_^−^ is a conjugate base of H_2_O_2_, therefore at acidic pH there is more H_2_O_2_ production. Although the energetic electrons of the NPP react with H_2_O to form H^•^ and OH^•^, these extremely reactive chemical species strongly react with bacteria. However, due to water absorption the negative ions are not believed to react with bacteria directly^46^. So in this condition the plasma induced chemistry in liquid plays an important role. In addition, we have proposed the effect of NPP treatment in form of equation (25-39), as previously discussed also^53^.


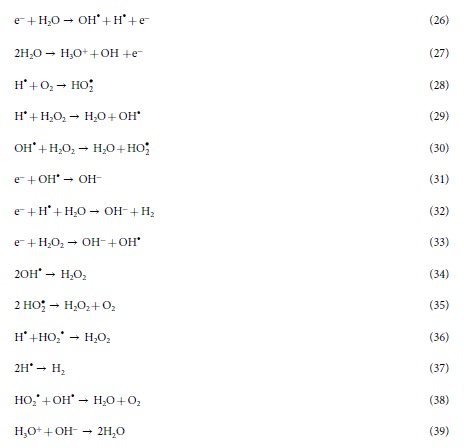


### Role of combined effect of the RS, pH and shock waves on the inactivation of bacteria

According to our recently published work the lifetime of OH^^•^^ ≈ 2.7 μs, NO ≈ 1.2 μs, H_2_O_2_ ≈1.4 μs and O_2_^•−^ ≈1.3 μs at 2 mm above the water surface in ambient air^54^. Moreover, the life time of ONOO^−^ is 0.2 s at pH 5 as reported earlier^46^. The antibacterial effect of ONOO^−^ and ONOOH are significant despite their lower half-life. ONOO^−^ is toxic to cells because of its ability diffuse through the cell wall before reacting. On the other hand, the ONOOH is a powerful oxidant for biological molecules^19^,^46^. The O_2_^•−^ is not major contributor because it convert to 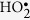 at low pH and also because most cells contain superoxide dismutase that convert O_2_^•−^ to O_2_ and H_2_O_2_^46^. 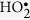 can play an important role in bacteria inactivation because of its ability to penetrate the cell membrane^46^. Therefore, the inactivation of *S. aureus* with both plasma sources occurs with almost the same radicals, although the amount can vary from plasma source to plasma source. The role of the pH is only to increase the reactivity of the radicals, and the pH has no direct effect on the bacterial inactivation in our studies. In addition, the temperature has no role in the inactivation of *S. aureus*. The shock waves play an important role in the inactivation, which creates a difference in the efficiency of NPP and Ar-DBD treatments. Additionally, the UV and x-rays are also generated during the NPP treatment that can also help in deactivation of *S. aureus*. However, the SEM images of the electrodes show that there is no degradation of electrode after the discharge that results in no nanoparticle formation in the solution.

### Conclusion

We finally concluded that 1) only pH and temperature has no significant role in bacteria inactivation; 2) ozone has role in bacteria killing but in our experiment ozone is not major factor; 3) combined action of RS and pH has important role in bacterial inactivation; 4) Shockwaves generated in NPP may also play significant role in bacterial inactivation; 5) XPS and MD simulation results show that PG is oxidized due to plasma action that may help in inactivation of *S. aureus*. Therefore, research on plasma-liquid interactions is a recommended field for further new advances in plasma medicine.

## Experimental Section

### Materials

Tryptic soy agar and typtic soy broth were purchased from MB cells (Seoul, Korea). *S. aureus* 1621(wild type), 11812 (penicillin resistant), 40510 (methicillin resistant) and 40512 (gentamicin resistant) were procured from KCCM (Korean Culture Center of Microorganisms, South Korea) and were used in the experiments. The H_2_O_2_ was measured using an titanyl ion^54^,^55^, and an NO^56^ detected using 4-amino-5-methylamino-2′,7′-difluorofluorescein (DAF-FM) as per given procedure in early research work. H2DCFDA (2’,7’-dichlorodihydrofluorescein diacetate; Invitrogen) was used to detect the intracellular ROS. OH was also measured using terephthalic acid (20 mM) soluble in saline was exposed to plasma (NPP or Ar-DBD) and the fluorescence of treated solutions was measured at 310/425 (ex./em.) nm^54^. OH and H_2_O_2_ scavenger 6-hydroxy-2,5,7,8-tetramethylchroman-2-Carboxylic Acid (Trolox) and NO scavenger 2- to 4-carboxyphenyl-4,4, 5,5-tetramethylimidazoline-1-oxyl-3-oxide (cPITO) were purchased from Sigma Aldrich. The RNA extraction was performed using an RNA extraction kit (Rneasy Mini Kit, Qiagen). cDNA synthesis was performed using the ReverTra Ace qPCR RT Master Mix with gDNA Remover kit (TOYOBO, Japan), and quantitative PCR was performed by using a Thunderbird Sybr® qPCR Mix kit (TOYOBO, Japan). The β-lactamase activity experiment was performed using a Beta-Lactamase Activity Colorimetric Assay Kit (BioVision, USA). Ozone is generated using ozone machine of Model: LAB-II, Company: OZONE TECH. Shock waves are measure using the piezoelectric pressure gauge of PCB PIEZOTRONICS Model 480C02.

### APP devices used for the experiments

We used a Marx generator to generate the nanosecond-pulsed plasma in liquid. This device is the simplest and most widely used for high-voltage pulse generation, as shown in the schematic diagram in Fig. 1a. Detailed descriptions are given in our previous work^13^. However, we used a pin-to-pin Pt electrode for this study. The peak voltage is 17 kV and peak current is 1 kA, with breakdown voltage of 6 kV and breakdown current of 0.7 kA. The product of the breakdown voltage and breakdown current was integrated with respect to the time required to reveal breakdown energy of 0.06 J/discharge.

The Ar-DBD plasma device consists of electrodes, dielectric layer (silicon dioxide (SiO_2_)) and hydration prevention layers aluminum oxide (Al_2_O_3_), and a magnesium oxide (MgO) layer. The electrode is 5 μm thick and 200 μm wide with a 200 μm electrode gap and MgO layer of ≈1 μm thick. The thickness of the SiO_2_ dielectric layer is about 30 μm and the diameter of the plasma discharge area is about 60 mm. To prevent hydration during plasma discharge, Al_2_O_3_ was added below the MgO layer. To generate plasma, Ar gas was injected into the device with l lpm flow rate. A commercial transformer for neon light is operated at 60 Hz as the AC power supply. The input voltage was about 70 V (V_rms_ is 0.6 kV and I_rms_ is 14 mA), the frequency is about the 22 kHz (Fig. 1b).

### Modeling section

As mentioned above, the computational details of the reactive MD simulations used in this work were presented in our previous studies^36^,^37^. Here only a brief explanation is given, focusing attention on the structure of the PG in our model and the impacts of the plasma species. We use the structure of the *S. aureus* PG as a model system. Its chemical structure and schematic representation can be found in ref. 36. The PG model system is composed of two disaccharides (i.e., two N-acetylglucosamine and N-acetylmuramic acid) with tetrapeptide (i.e., l-alanine-d-*iso*-glutamine-l-lysine-d-alanine) stems, connected with one pentaglycine interpeptide. With this construction we are able to take into account all possible atomic bonds in the PG structure^36^. We employ the ReaxFF glycine-force field^38^ in our MD simulations to study the interaction of the reactive (i.e., O, OH, O_3_ and H_2_O_2_) as well as non-reactive (i.e., O_2_, H_2_O) plasma species with PG of *S. aureus*.

The PG structure is placed in a box with dimensions ~75 Å × 88 Å × 51 Å without periodic boundary conditions. Prior to the particle impacts, the PG structure is equilibrated at room temperature (i.e., 300 K). In all simulations (i.e, both during the thermalization as well as during the particle impact simulations), we use a time step of 0.1 fs. To study all possible damaging mechanisms of PG and to get statistically valid results for bond-breaking processes, we perform 50 runs for each impinging species. In all simulations, 10 incident particles of a single species (e.g., 10 O_3_ molecules) are initially randomly positioned at a minimum distance of 10 Å around the PG structure and from each other in order to prevent interactions between the impinging particles and the PG structure prior to the actual simulation run. The initial energy of impinging plasma species corresponds to room temperature and their velocity directions are chosen randomly. Every simulation trajectory lasts 300 ps; we carefully checked that this time is long enough to obtain a chemically destructed PG structure, at least if a critical bond in the structure is broken. Thus, at the end of the simulation all plasma species interacted with the structure, possibly resulting in the breaking of various bonds.

### OES spectra measurement of the APP

The spectra of the NPP and DBD emissions were recorded using an HR4000CG-UV-NIR (Ocean Optics, FL, USA) over a wide wavelength range from 200 to 1100 nm to cover all of the experimental conditions explored in this study.

### Temperature measurement and pH

After exposure, the pH and temperature of the sample were measured using a pH meter (Eutech Instruments, Singapore) and an Infrared (IR) camera (Fluke Ti100 Series Thermal Imaging Cameras, UK).

### Sample preparation, viability assay and morphology

All strains of *S. aureus* (wild, penicillin, methicillin, and gentamicin) were cultured in TSB culture media until they reached the logarithmic growth phase. The cell suspension was centrifuged, and the supernatant was removed. The pellet was re-suspended in sterile 0.8% saline. All bacterial samples were suspended and were then placed into a sample container for the NPP treatment and in a Petri dish of 90 mm in size for the DBD treatment. In the case of the DBD treatment, the distance between the outer electrode and the solution was kept at 5 mm during exposure. After exposure to NPP and DBD, serial dilutions of 10^7^, 10^6^, 10^5^, 10^4^, and 10^3^ CFU/ml were prepared. The samples were mixed thoroughly, and 100 μl of each of the plasma-treated solutions was transferred and spread uniformly on TS agar culture medium in a standard Petri dish (90 mm). These samples were then sealed and incubated at 37 °C for about 16 h to count the CFUs. In this study, a relative reduction of the control was used to represent the inactivation efficacy, where the control CFU was defined as one (unit) for normalization. Analyses were conducted with a SEM (JSM 7001 F, JEOL, Tokyo, Japan) to examine the morphology of the cells. Briefly, all bacterial samples exposed to NPP and DBD were fixed in 1 mL of Karnovsky’s fixative (2% paraformaldehyde and 2% glutaraldehyde) overnight, as described in previous reports^57^. The SEM sample preparation involved dehydration of the material in hexamethyldisilazane (HMDS), followed by mounting and coating on glass with carbon tape and an examination via FESEM.

### Measurement of the ROS and RNS

The ROS levels in saline were analyzed after the 4^th^ discharge of the NPP exposure and the 4^th^ discharges + Trolox/cPITO and DBD plasma for 5 min and 5 min + Trolox/cPITO (1 μM Trolox used for OH, H_2_O_2_ scavenger and 0.5 mM cPITO used for NO scavenger). Afterwards, the amount of OH, H_2_O_2_ and NO radical was determined. We have used a method from a previous report of our group^15^. The total ROS inside all bacterial strains are studied using the H2DCFDA. 10^7^ cells/mL of all bacterial samples in saline were exposed to the 4^th^ discharge of NPP and to 5 min of DBD plasma. Then, the samples were transferred to a microcentrifuge tube. All exposed bacterial samples were washed with PBS, and 500 ml of 10 Mm H2DCFDA were added. After incubation for 1 h at 30 °C, the cells were washed with PBS twice. Then, the bacterial cells were recovered with PBS at 30 °C for 30 min and analysed at 495/515 (ex./em.) nm using a microplate reader. The mean fluorescence intensity was determined at the corresponding excitation and emission wavelengths.

### RNA extraction for quantitative real time PCR

To perform a quantitative evaluation of MRSA related gene expression. Firstly, culture all strain containing antibiotic (MRSA, PRSA, and GRSA) in TSB culture media respectively. Whereas, wild type *S. aureus* was culture without containing antibiotics. After overnight culture the cell suspension was centrifuged, and the supernatant was removed. The pellet was re-suspended in sterile 0.8% saline. All bacterial samples were suspended and were then exposed with 4^th^ discharge of NPP and 5 min of DBD plasma. After exposure, total RNA was extracted from treated and untreated samples of all four strains of S.aureus using an RNeasy Mini Kit, and these were converted to cDNA using reverse transcriptase and random primers (GoScriptTM Reverse Transcription System, Promega). The same amount of total RNA was used for the cDNA synthesis (Take3, Biotek). The resulting cDNAs were used for the qPCR analysis (CFX96, Biorad) with primers (Table S1, Macrogen) of 16s rRNA (the RNA component of the small subunit), MecA, MecI, MecRI, FemA, and 16s rRNA used as a house-keeping gene.

### Statistical analysis

All values are represented as mean ± SD of the indicated number of replicates. The statistical analyses of the data were performed using Student’s t-test to establish significance between the data points, and significant differences are based on P < 0.05 or P < 0.01.

## Additional Information

**How to cite this article**: Hoon Park, J. *et al*. A comparative study for the inactivation of multidrug resistance bacteria using dielectric barrier discharge and nano-second pulsed plasma. *Sci. Rep*. **5**, 13849; doi: 10.1038/srep13849 (2015).

## Supplementary Material

Supplementary Information

## Figures and Tables

**Table 1:** Experimental composition of bacteria with or without plasma treatment obtained using XPS.

Bacteria	%C	%O
*S. aureus* (wild type)_control	44.95	39.97
*S. aureus* (wild type)_NPP	31.55	52.51
*S. aureus* (wild type)_DBD	35.70	45.48
PRSA_control	52.78	32.99
PRSA_NPP	42.98	41.15
PRSA_DBD	45.93	38.98
MRSA_control	49.34	36.85
MRSA_NPP	45.25	39.50
MRSA_DBD	47.25	38.62
GRSA_control	47.64	38.50
GRSA_NPP	41.69	40.04
GRSA_DBD	45.93	39.98
