# Supplementary material for: A comparative study for the inactivation of multidrug resistance bacteria using dielectric barrier discharge and nano-second pulsed plasma
Source: Sci Rep. 2015 Sep 9;5:13849. doi: 10.1038/srep13849 (PMC4563361; doi:10.1038/srep13849)
Supplement: Supplementary Information [file srep13849-s1.pdf]

A comparative study for the inactivation of multidrug resistance bacteria using dielectric barrier discharge and nano-second pulsed plasma

Ji Hoon Park[1], Naresh Kumar[1], Dae Hoon Park[1], Maksudbek Yusupov[2], Erik C. Neyts[2], Christof C. W. Verlackt[2], Annemie Bogaerts[2], Min Ho Kang[1], Han Sup Uhm[1], Eun Ha Choi[1], Pankaj Attri[1]

[1]*Plasma Bioscience Research Center / Department of Electrical and Biological Physics, Kwangwoon University, Seoul, Korea 139-701.*
[2]*Research Group PLASMANT, Department of Chemistry, University of Antwerp, Universiteitsplein 1, 2610 Antwerp, Belgium*

Figure S1: (a) Voltage and current graph for NPP; (b) Voltage and current graph for Ar-DBD; (c) Temperature and (d) pH of the saline solution after the 4th discharge of NPP and 5 min Ar-DBD treatment. All values are expressed as ± SD in triplicates.

Figure S2: Determination of (a) H_2O_2 concentration, (b) OH radical, (c) NO radical in 1 ml saline after the 4th discharge of NPP and 5 min Ar-DBD treatment. All values are expressed as ± SD in triplicates. Students't-test was performed to control (* denotes P<0.05 and ** denotes P<0.01)

Figure S3: Shockwaves analysis by calculating the voltage as function of time.

Figure S4: SEM image of the Penicillum-resistant, Methicillin-resistant and Gentamicin-resistant S. aureus bacteria after the 4th discharge of NPP and 5 min Ar-DBD treatment.

Figure S5: C 1s XPS data of the wild type S. aureus (a) control, (b) after the 5 min Ar-DBD treatment and (c) after the 4th discharge of NPP.

Figure S6: O 1s XPS data of the wild type S. aureus (a) control, (b) after the 5 min Ar-DBD treatment and (c) after the 4th discharge of NPP.

Figure S7: C 1s XPS data of the Penicillum-resistant S. aureus (a) control, (b) after the 5 min Ar-DBD treatment and (c) after the 4th discharge of NPP.

Figure S8: O 1s XPS data of the Penicillum-resistant S. aureus (a) control, ((b) after the 5 min Ar-DBD treatment and (c) after the 4th discharge of NPP.

Figure S9: C 1s XPS data of the Methicillin-resistant S. aureus (a) control, (b) after the 5 min Ar-DBD treatment and (c) after the 4th discharge of NPP.

Figure S10: O 1s XPS data of the Methicillin-resistant S. aureus (a) control, (b) after the 5 min Ar-DBD treatment and (c) after the 4th discharge of NPP.

Figure S11: C 1s XPS data of the Gentamicin-resistant S. aureus (a) control, (b) after the 5 min Ar-DBD treatment and (c) after the 4th discharge of NPP.

Figure S12: C 1s XPS data of the Gentamicin-resistant S. aureus (a) control, (b) after the 5 min Ar-DBD treatment and (c) after the 4th discharge of NPP.

Figure S13: Inactivation of wild type and multidrug resistance bacterial strains of S. aureus by the different pH solution. All values are expressed as ± SD in triplicates.

Figure S14: Inactivation of wild type and multidrug resistance bacterial strains of S. aureus by the Ozone treatment for 30 s (inside the solution and 10 mm above the solution). All values are expressed as ± SD in triplicates.

Figure S15: Amount of Ozone produced for 5 min in Ar-DBD. All values are expressed as ± SD in triplicates.

Figure S16: Inactivation of wild type and multidrug resistance bacterial strains of S. aureus by the pH 4 solution with 50 μM H_2O_2. All values are expressed as ± SD in triplicates.

Table S1. Primers used in expression analysis

Name	Forward sequence	Reverse sequence
MecA	ATGAGATTAGGCATCGTTCC	TGGATGACAGTACCTGAGCC
MecI	CTGCAGAATGGGAAGTTATG	TTACAAGTGAATTGAAACCGCAGA
mecRI	CAAAAGCACCGTTACTATCTGC	GAGTAAATTTTGGTCGAATGCTA
femA	ACAATGATGGCGAGATTACAGGA	ACC GCTAAAGGTACTAACAACAT
16S	AGAGCAAGCGGACCTCATAA	TTCATGGAGTCGAGTTGCAG

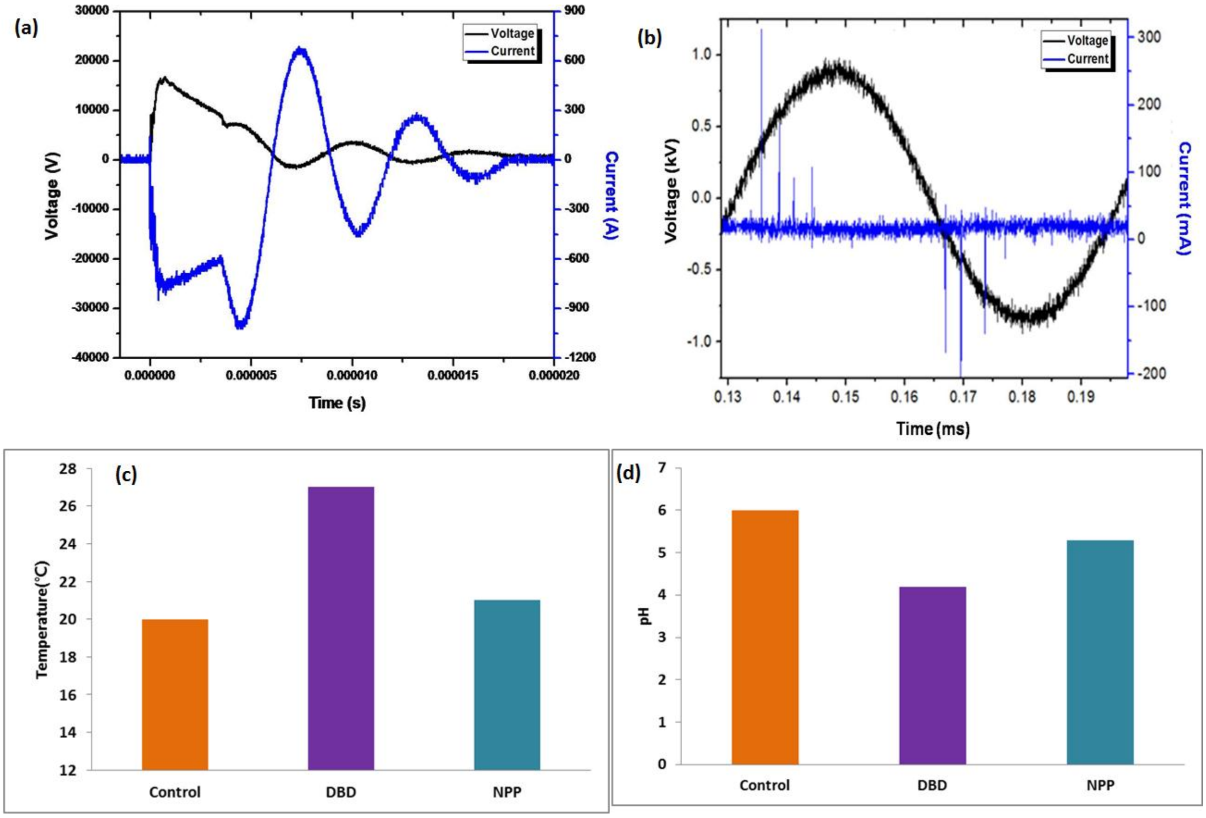

Figure S1

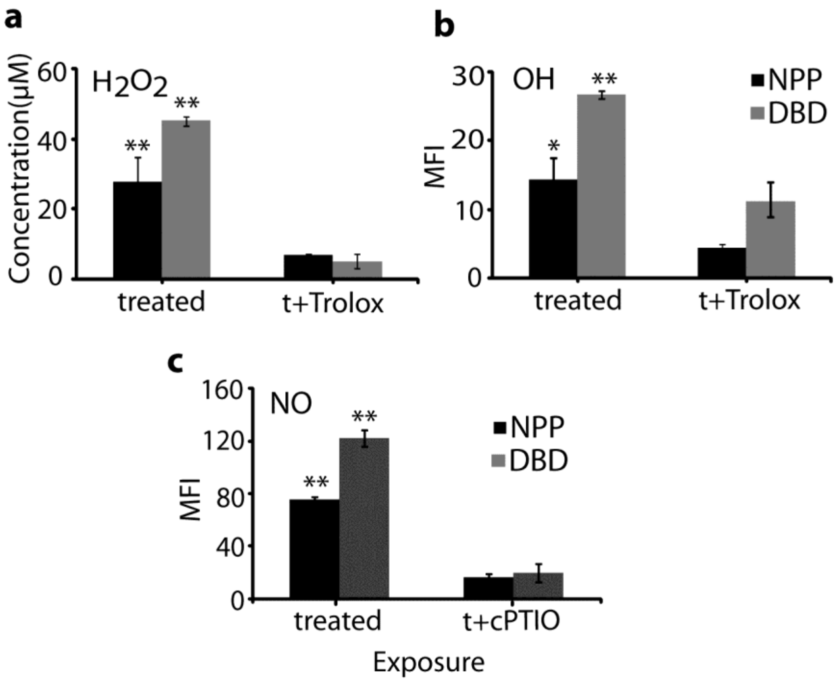

Figure S2

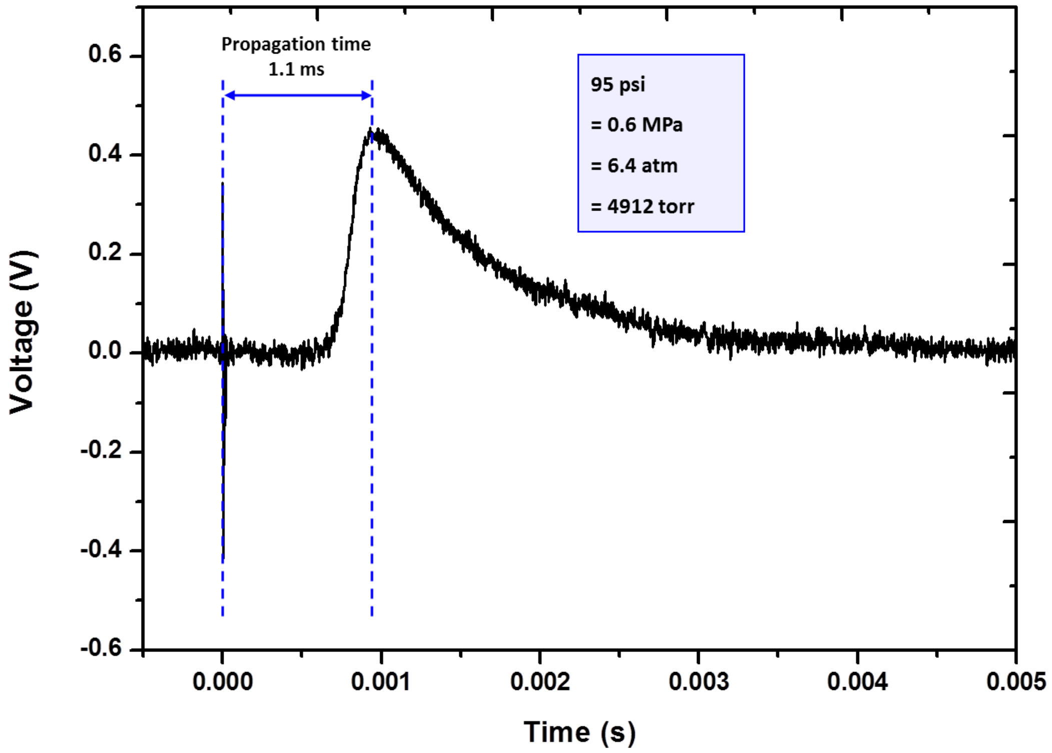

Propagation time
1.1 ms
95 psi
= 0.6 MPa
= 6.4 atm
= 4912 torr
0.6
0.4
0.2
0.0
-0.2
-0.4
-0.6
Voltage (V)
0.000
0.001
0.002
0.003
0.004
0.005
Time (s)

Figure S3

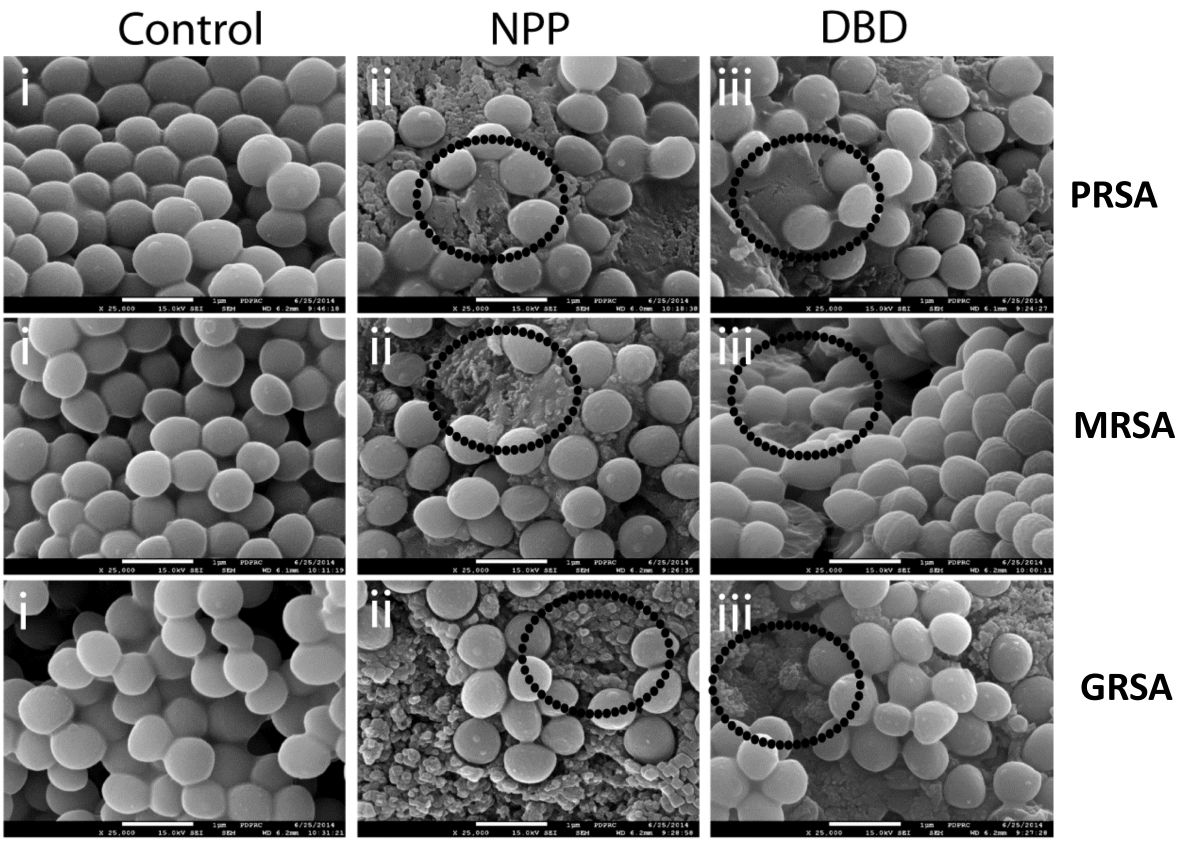

Figure S4.

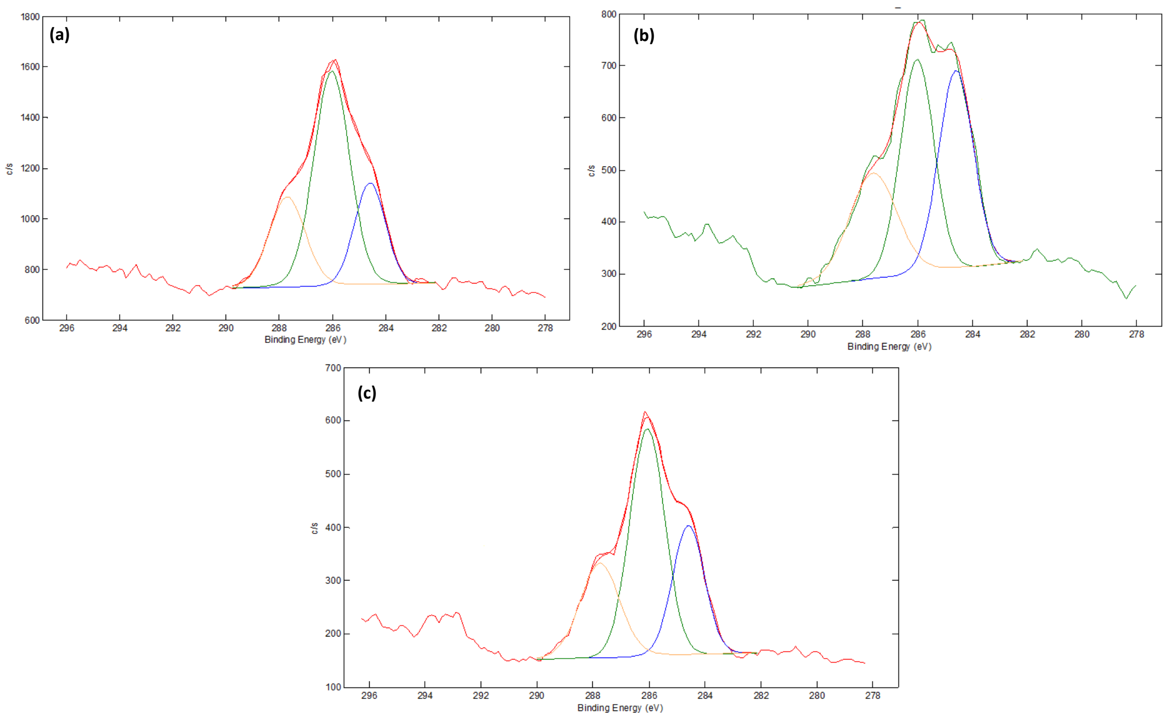

Figure S5.

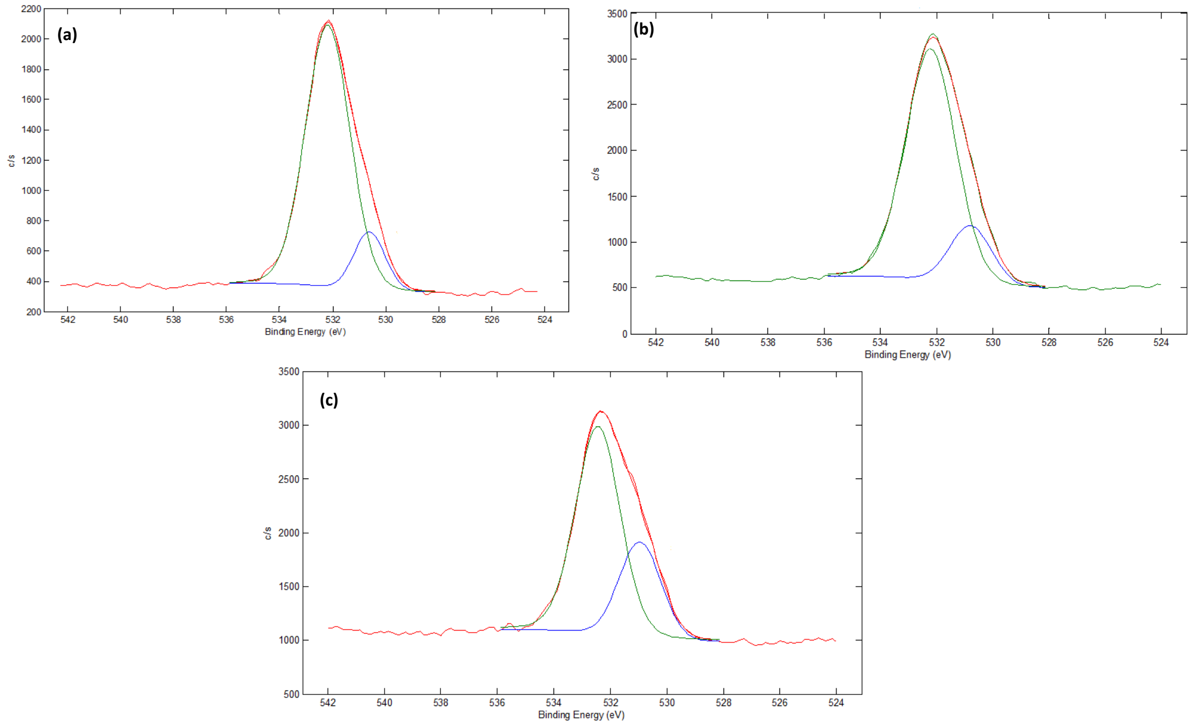

Figure S6.

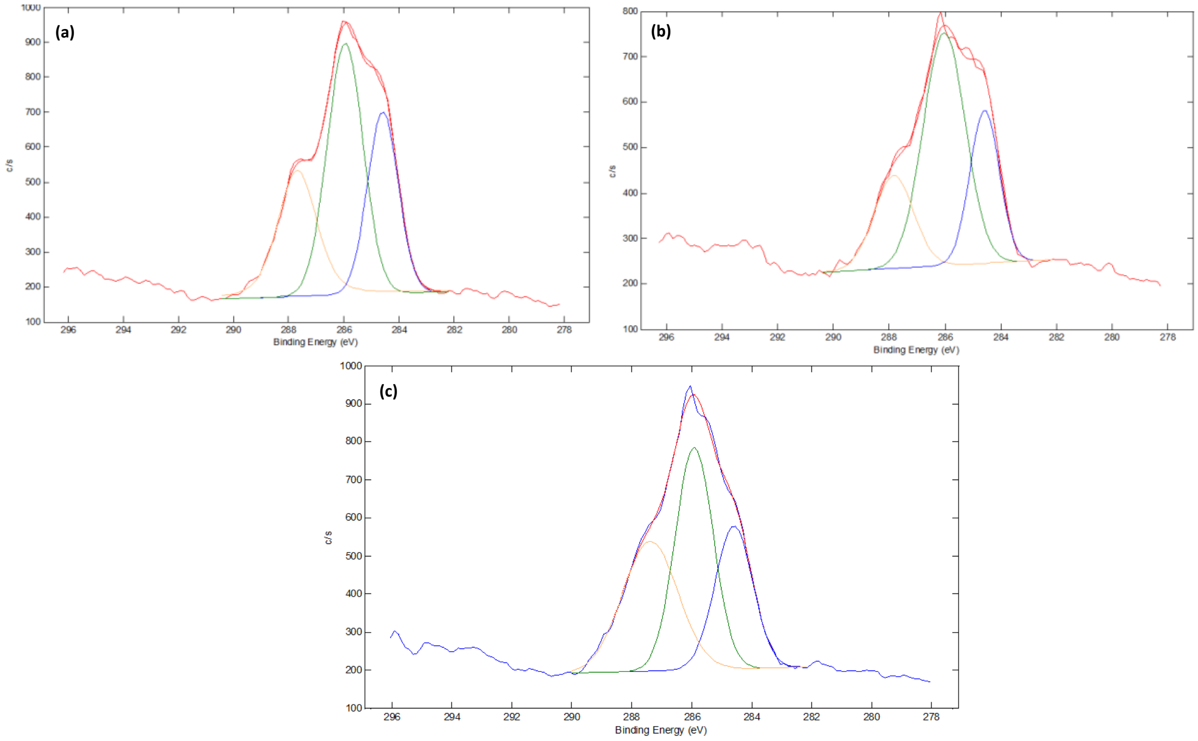

Figure S7.

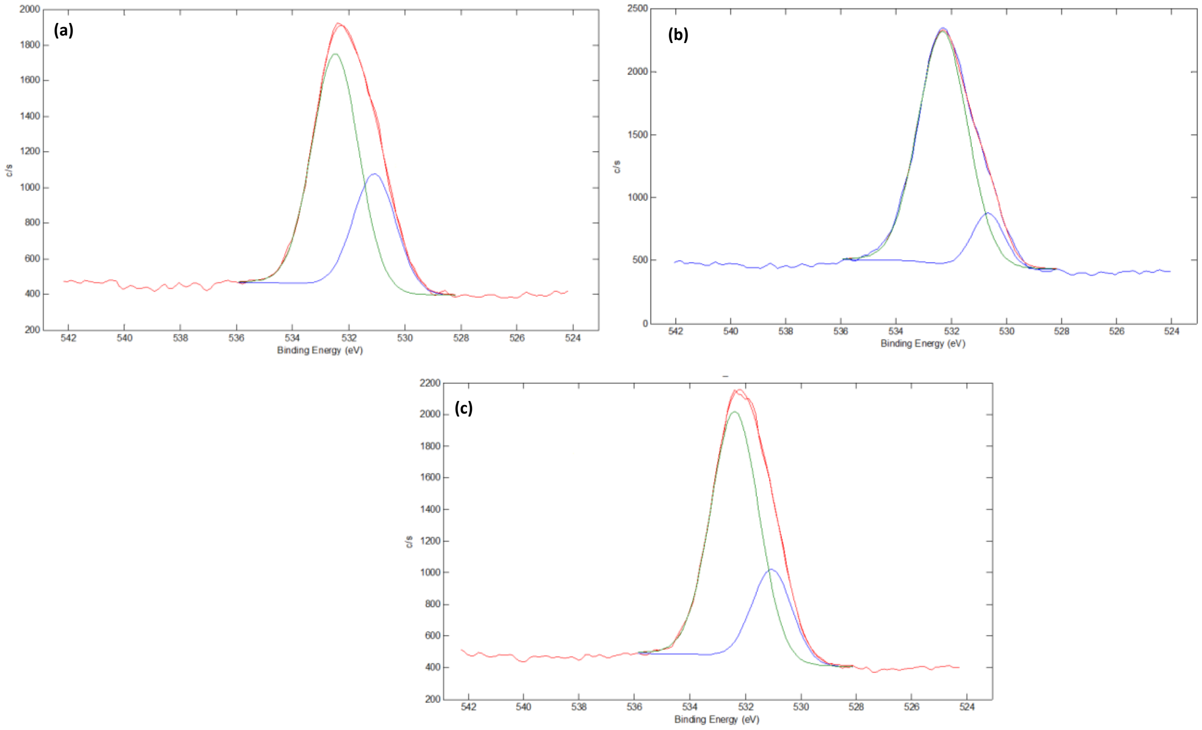

Figure S8.

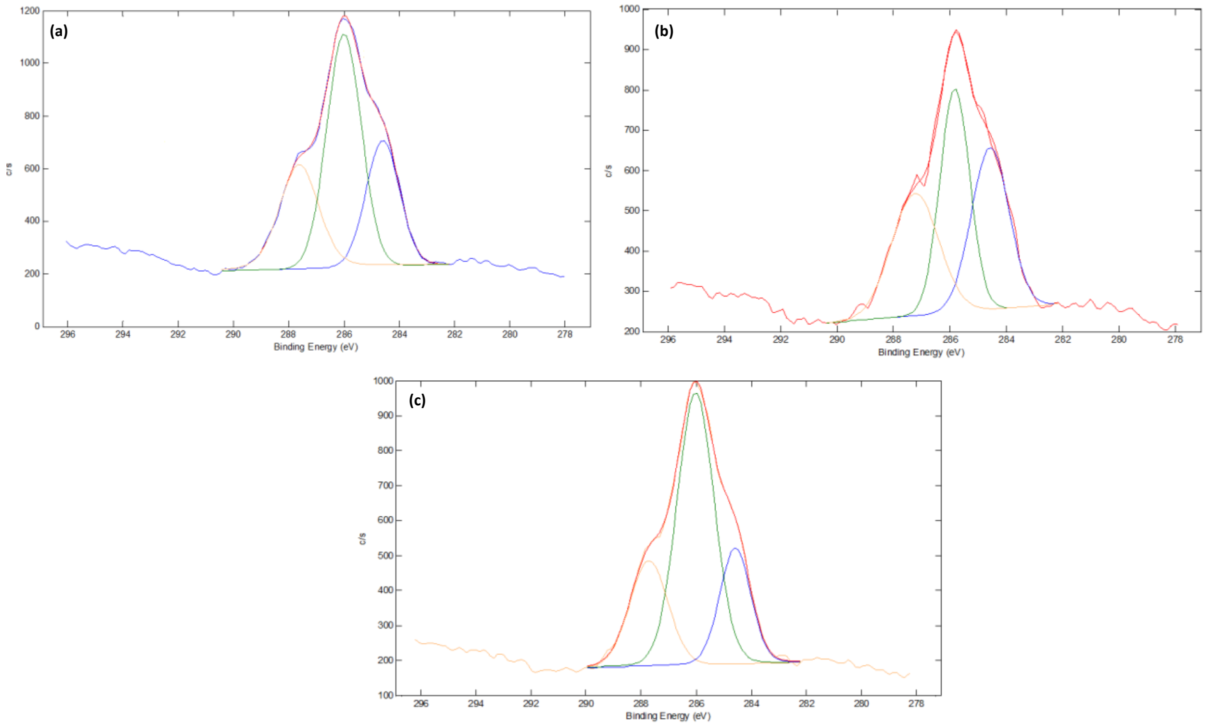

Figure S9.

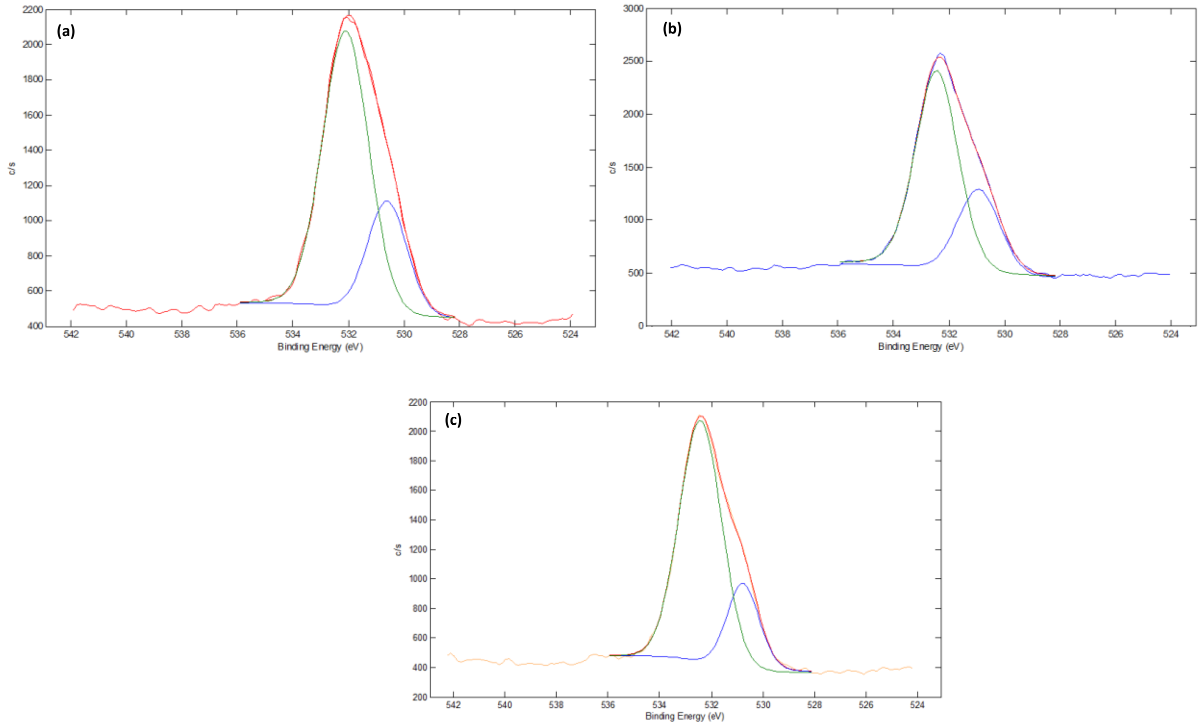

Figure S10.

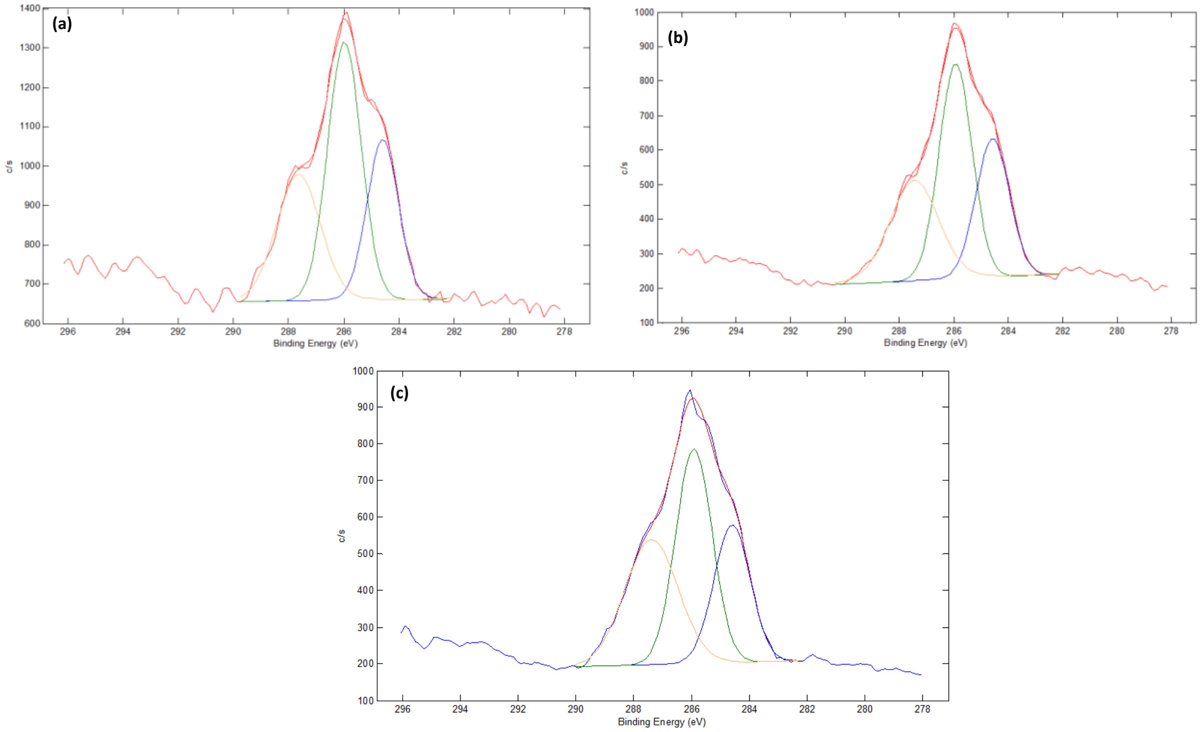

Figure S11.

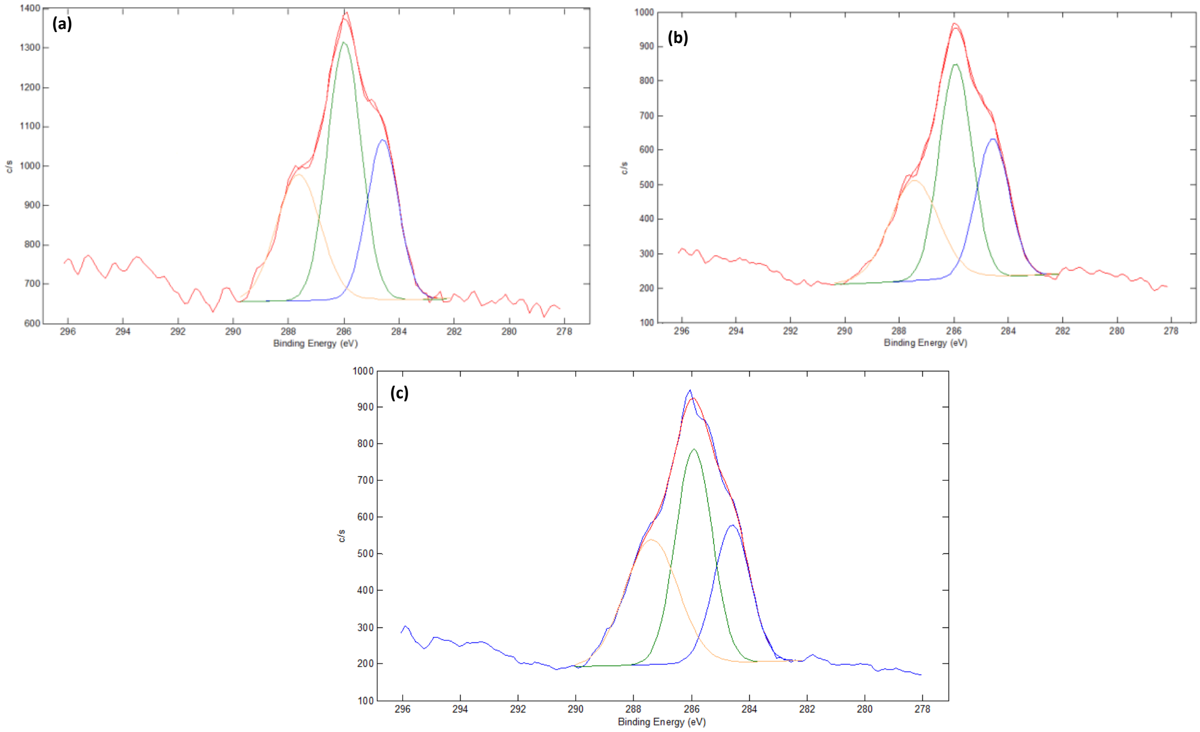

Figure S12.

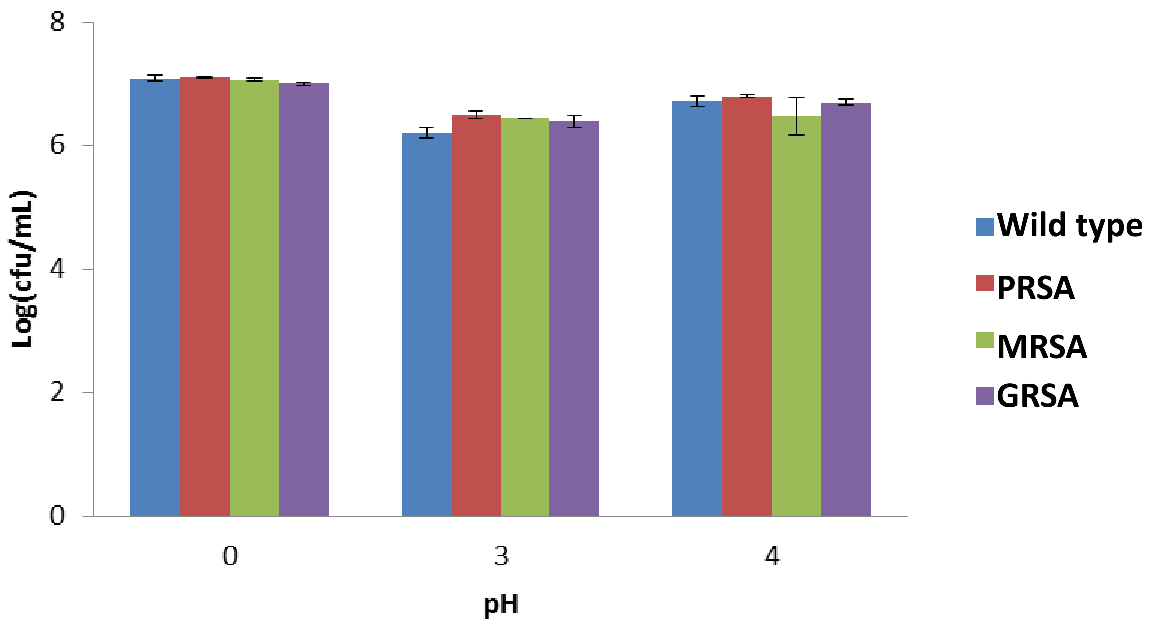

Figure S13.

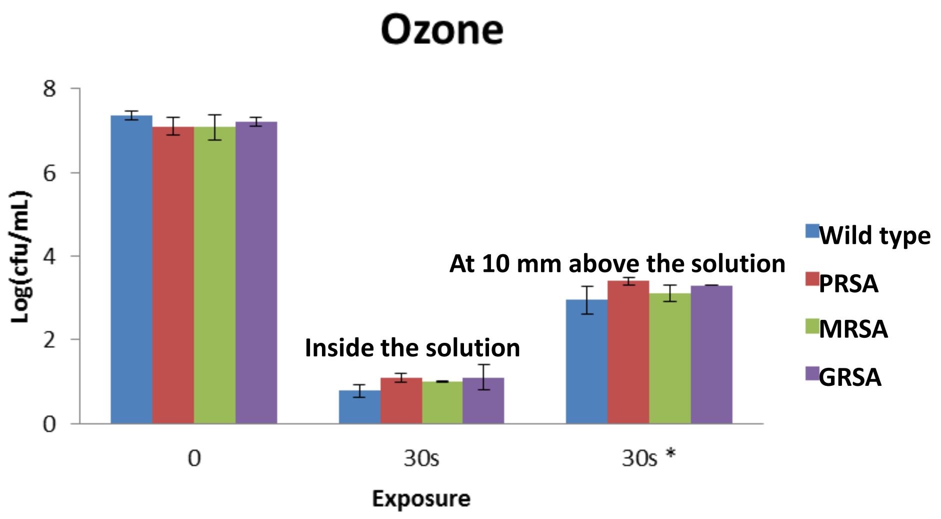

Figure S14.

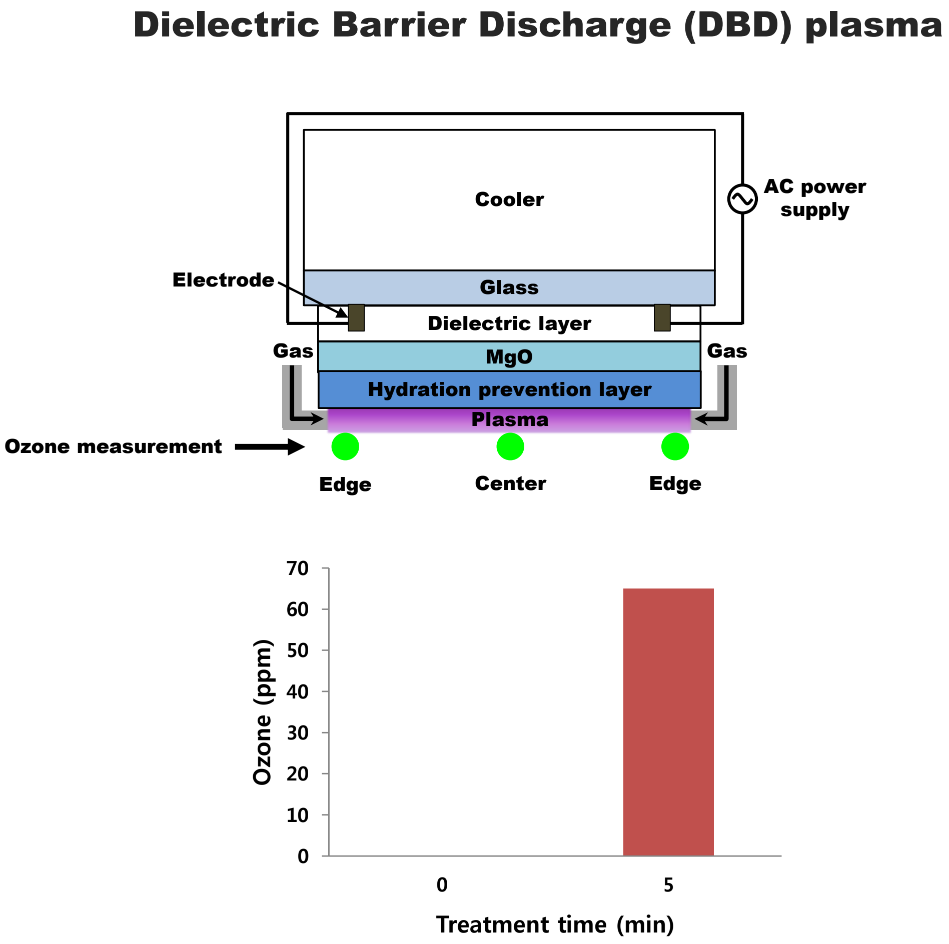

Figure S15.

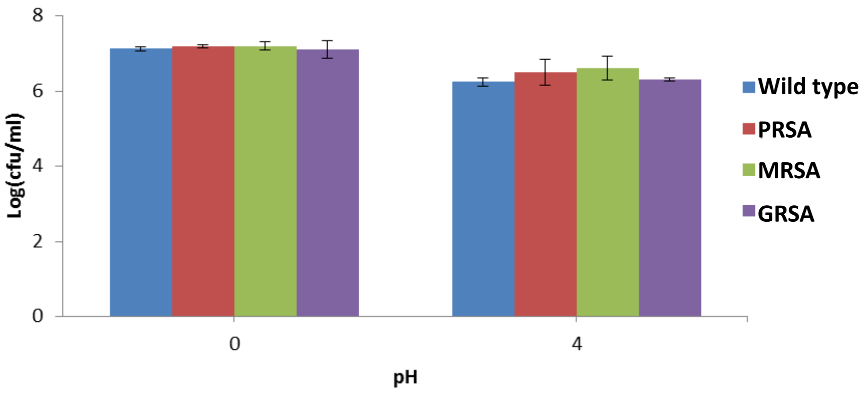

Figure S16.
